# Aging “Well” in Place: A Qualitative Case Study That Explores the Influence of a Digital Equity Project

**DOI:** 10.1093/geroni/igaf122.2594

**Published:** 2025-12-31

**Authors:** Josie Santilli, Skye Leedahl, Jill Juris, Erica Liebermann, Casey McGregor

**Affiliations:** University of Rhode Island, Kingston, Rhode Island, United States; University of Rhode Island, Kingston, Rhode Island, United States; Appalachian State University, Boone, North Carolina, United States; University of Rhode Island, Kingston, Rhode Island, United States; University of Rhode Island, Kingston, Rhode Island, United States

## Abstract

Older adults prefer to age in the place they call home. However, home is changing as technology impacts daily living. The ability to age well while aging in place (AIP) may no longer be sustainable without digital equity. The purpose of this qualitative case study, guided by the Ecological Theory of Aging, was to explore how advancing digital equity through the iPad Project influenced older adults’ ability to AIP. The participatory design of this case study sought to learn from older adults who had completed the iPad Project and crossed the digital divide and from collaborators who designed, implemented, and evaluated the intervention. A comprehensive document review was completed and semi-structured interviews were conducted with 30 older adult project participants and collaborators to explore participant experiences since completing the project. Their responses were analyzed using thematic analysis. Themes identified were expanded technology acceptance and adoption, enhanced quality of life, and increased access to healthcare. The findings suggest that AIP extends beyond the location where one lives. A contemporary perspective reflective of our digital world considers the lifestyle for how older adults live to age “well” in place (AWIP). The iPad Project positively influenced participants’ lives by supporting their goal of AWIP.

